# Context‐Dependent Temporal Changes in Hypnotics Involved in Suicide Attempts

**DOI:** 10.1002/npr2.70126

**Published:** 2026-04-30

**Authors:** Taro Sasaki, Norio Sugawara, Motoharu Furukawa, Hirotaka Iwaki, Shiro Suda, Norio Yasui‐Furukori

**Affiliations:** ^1^ Department of Psychiatry Dokkyo Medical University School of Medicine Mibu Tochigi Japan; ^2^ Department of Psychiatry Jichi Medical University Shimotuke Tochigi Japan; ^3^ Department of Psychiatry Hachinohe City Hospital Hachinohe Aomori Japan

**Keywords:** benzodiazepines, orexin receptor antagonists, self‐poisoning, suicide attempt, temporal trend

## Abstract

**Background:**

Recent prescribing practices have shifted from benzodiazepines (BZs) toward non‐GABAergic hypnotics, including dual orexin receptor antagonists (DORAs) and melatonin receptor agonists (MRAs). We examined whether hypnotics involved in suicide attempts changed over time in a context‐dependent manner.

**Methods:**

We conducted a multicenter retrospective cohort study of consecutive patients presenting with suicide attempts at three hospitals in Japan between April 2020 and March 2025. Hypnotics involved in attempts were identified from empty medication packages collected at presentation. Annual proportions of BZs and non‐GABAergic hypnotics (OMs: DORAs and MRAs) were analyzed using Cochran–Armitage trend tests under three conditions: (1) all suicide attempts, (2) overdose‐related attempts, and (3) overdose‐related attempts involving a hypnotic. Additional analyses separated DORAs from MRAs.

**Results:**

Among 1111 suicide attempt encounters, 648 were overdose‐related. OM involvement increased significantly over time across all denominators. In contrast, BZ involvement declined significantly only among overdose‐related attempts. When OMs were disaggregated, DORA involvement showed a significant upward trend using overdose‐related attempts as the denominator (*χ* ^2^ = 7.3048, *p* = 0.006877) and using all suicide attempts as the denominator (*χ* ^2^ = 7.6384, *p* = 0.005714). MRA (ramelteon) involvement did not show significant temporal change in either analysis. Overall, the increase in OM involvement was primarily attributable to DORAs.

**Conclusion:**

Hypnotics involved in suicide attempts changed in a context‐dependent manner during the study period. The increase in non‐GABAergic hypnotics was driven by DORAs, whereas reductions in BZ involvement were detectable only in overdose‐related contexts. These findings suggest that evolving hypnotic availability may influence the profile of medications involved in self‐poisoning.

## Abstract 2

Temporal changes in hypnotics involved in suicide attempts showed context‐dependent shifts. Dual orexin receptor antagonists increased significantly, while benzodiazepine involvement declined only in overdose‐related attempts. Melatonin receptor agonists showed no significant trend during 2020.

## Graphical abstract

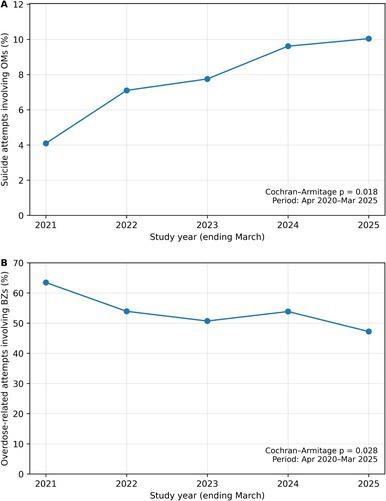

## 1 Introduction

Medication overdose is one of the most common methods of suicide attempts, and the drugs ingested are strongly shaped by what is readily available as prescribed or over‐the‐counter medications. In emergency cohorts, psychotropics—including hypnotics, anxiolytics, antidepressants, and antipsychotics—are repeatedly reported as major contributors to self‐poisoning presentations [1]. Among hypnotics, benzodiazepine receptor agonists (BZs) have been widely used for decades; however, concerns about dependence, cognitive impairment, and toxicity in overdose have led to increasing caution [2, 3, 4]. Importantly, benzodiazepines differ in their relative toxicity, and some agents appear to be associated with more severe outcomes in overdose compared with other sedative drugs [2, 3, 4].

Dual orexin receptor antagonists (DORAs) and melatonin receptor agonists (MRAs) have emerged as non‐GABAergic alternatives for insomnia, with established efficacy in randomized trials and growing use in routine practice [5, 6, 7]. These agents are generally considered to have a favorable safety profile, with limited respiratory depression and fewer concerns regarding tolerance and withdrawal than GABAergic hypnotics, which may be particularly relevant when treating patients at risk of intentional overdose [5, 6, 7, 8].

Despite these developments, real‐world data describing temporal changes in hypnotic agents involved in suicide attempts remain limited. Patterns are likely to be context dependent: within overdose‐related attempts, the medications used reflect those readily available at home, which may change over time with shifts in insomnia pharmacotherapy and risk‐sensitive prescribing. To address this gap, we examined temporal changes in the involvement of BZs and non‐BZ hypnotics (dual orexin receptor antagonists and melatonin receptor agonists; hereafter OMs) among consecutive patients presenting with suicide attempts from April 2020 through March 2025, using two denominators to capture overall and overdose‐related contexts.

## 2 Methods

### 2.1 Study Design and Cohort

This retrospective multicenter cohort study included consecutive patients who presented with suicide attempts between April 2020 and March 2025 and underwent psychiatric assessment at one of three tertiary‐care hospitals in Japan: Dokkyo Medical University Hospital, Jichi Medical University Hospital, and Hachinohe City Hospital. Study years were defined as April through March and labeled by the ending calendar year (2021–2025). Suicide attempts were identified based on clinical documentation at presentation. This study was conducted within the framework of the DJ project, a structured suicide‐prevention‐oriented clinical framework that has been described previously [9]. The DJ project uses harmonized clinical assessment procedures and standardized data abstraction across participating sites, allowing pooled analyses.

Among these patients, overdose‐related attempts were defined as intentional ingestion of medications in excess of prescribed or recommended doses, including both prescription and over‐the‐counter agents. For overdose‐related attempts, medications were identified by reviewing empty medication packages (e.g., blister packs or bottles) collected at presentation and documented in the medical record. Because hypnotic involvement was ascertained from empty medication packages, involvement of hypnotics was evaluated in attempts that included medication ingestion (self‐poisoning). If an attempt involved multiple methods including medication ingestion, it was classified as overdose‐related.

Analyses using different denominators were prespecified to explore context‐dependent patterns rather than to imply mutually exclusive patient groups.

### 2.2 Classification of Hypnotics

Hypnotics of interest were classified into two categories:

1. benzodiazepine receptor agonists (BZs; benzodiazepines and non‐benzodiazepine Z‐drugs), and
2. orexin receptor antagonists and melatonin receptor agonists (OMs).

In a supplementary analysis, OMs were further subdivided into dual orexin receptor antagonists (DORAs) and melatonin receptor agonists (MRAs; ramelteon). Because a single suicide‐attempt encounter could involve multiple hypnotic agents, DORA and MRA counts were summarized descriptively at the agent level and may exceed the number of OM‐involved encounters. In addition, we examined encounter‐level involvement of DORAs and MRAs separately (presence/absence per encounter) using the Cochran–Armitage test for trend with the same denominators as the main analyses.

### 2.3 Statistical Analysis

Temporal trends in hypnotic involvement across the 5‐year study period were assessed using the Cochran–Armitage test for trend. Separate analyses were conducted using two denominators:

1. all suicide attempts, and
2. overdose‐related suicide attempts.

A two‐sided *p* < 0.05 was considered statistically significant. Statistical analyses were performed using R version 4.5.0 (R Foundation for Statistical Computing, Vienna, Austria).

## 3 Results

### 3.1 Temporal Trends in OM Involvement

**FIGURE 1:**
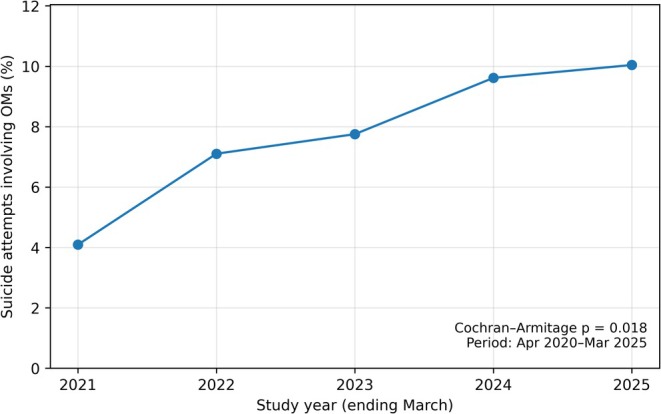
Temporal trends in involvement of orexin receptor antagonists and melatonin receptor agonists (OMs), identified from empty medication packages, among patients with suicide attempts at three hospitals in Japan from April 2020 through March 2025. Denominator: All suicide attempt encounters in each study year (ending March). The proportion of suicide attempt encounters involving OMs increased steadily over the study period (Cochran–Armitage test for trend, *p* = 0.018).

Overall, 1111 suicide attempt encounters were included, including 648 overdose‐related encounters (Table 1). The proportion of suicide attempt encounters involving OMs increased steadily over time (Figure 1). When all suicide attempts were used as the denominator, OM involvement rose from 4.1% (7/171) in 2021 to 10.0% (24/239) in 2025, with a significant upward trend (*p* for trend = 0.018). When overdose‐related attempts were used as the denominator, OM involvement increased from 6.7% (7/104) in 2021 to 16.7% (24/144) in 2025 (*p* for trend = 0.022).

**TABLE 1:** Annual numbers of suicide attempt encounters and hypnotic involvement (identified from empty medication packages).

Study year (ending March)	All suicide attempts, *n*	Overdose‐related attempts, *n*	OM involved, *n* (%) (all attempts)	OM involved, *n* (%) (overdose attempts)	BZ involved, *n* (%) (all attempts)	BZ involved, *n* (%) (overdose attempts)
2021	171	104	7 (4.1%)	7 (6.7%)	66 (38.6%)	66 (63.5%)
2022	183	102	13 (7.1%)	13 (12.7%)	55 (30.1%)	55 (53.9%)
2023	258	142	20 (7.8%)	20 (14.1%)	72 (27.9%)	72 (50.7%)
2024	260	156	25 (9.6%)	25 (16.0%)	84 (32.3%)	84 (53.8%)
2025	239	144	24 (10.0%)	24 (16.7%)	68 (28.5%)	68 (47.2%)
Total	1111	648	89 (8.0%)	89 (13.7%)	345 (31.1%)	345 (53.2%)

*Note:* Study years were defined as April through March and labeled by the ending calendar year.

Abbreviations: BZ, benzodiazepine receptor agonists; OM, orexin receptor antagonists and melatonin receptor agonists.

### 3.2 Temporal Trends in BZ Involvement

**FIGURE 2:**
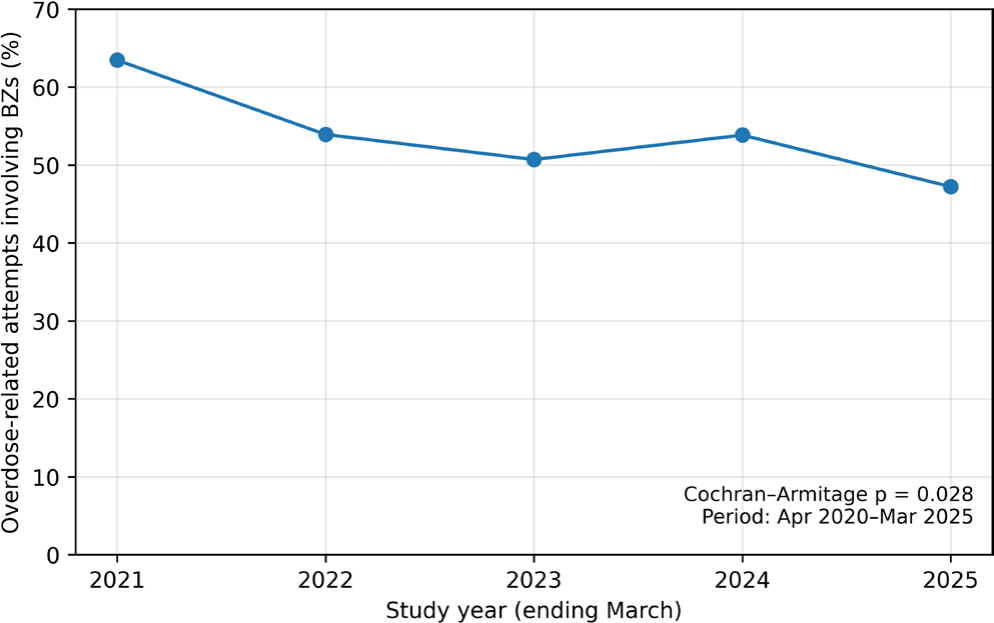
Temporal trends in benzodiazepine receptor agonist (BZ) involvement, identified from empty medication packages, among patients with overdose‐related suicide attempts at three hospitals in Japan from April 2020 through March 2025. Denominator: Overdose‐related suicide attempt encounters in each study year (ending March). When analyses were restricted to overdose‐related suicide attempts, a significant decreasing trend in BZ involvement was observed over time (Cochran–Armitage test for trend, *p* = 0.028).

Temporal trends in BZ involvement differed depending on the analytical denominator. When all suicide attempts were considered, the proportion of attempts involving BZs showed no significant trend over time (*p* for trend = 0.113). In contrast, when analyses were restricted to overdose‐related suicide attempts, BZ involvement decreased from 63.5% (66/104) in 2021 to 47.2% (68/144) in 2025, with a significant downward trend (*p* for trend = 0.028). This finding suggests that reductions in BZ involvement over time were specific to overdose‐related contexts (Figure 2).

Annual numbers of suicide attempt encounters and overdose‐related encounters are shown in Figure S1. In a supplementary breakdown of OMs, the number of DORA agents identified increased over time (7 in 2021, 8 in 2022, 19 in 2023, 24 in 2024, and 25 in 2025), whereas MRA agents (ramelteon) remained infrequent (0, 6, 3, 2, and 1, respectively) (Table S1; Figure S2). Overall, DORAs accounted for 87% (83/95) of OM agents identified during the study period. Consistent with this descriptive pattern, Cochran–Armitage trend tests demonstrated a significant increase in DORA involvement over time when overdose‐related attempts were used as the denominator (*χ* ^2^ = 7.3048, *p* = 0.006877) and when all suicide attempts were used as the denominator (*χ* ^2^ = 7.6384, *p* = 0.005714). In contrast, MRA involvement showed no significant temporal trend in either analysis (overdose‐related attempts: *χ* ^2^ = 0.90403, *p* = 0.3417; all suicide attempts: *χ* ^2^ = 0.84766, *p* = 0.3572).

## 4 Discussion

This study demonstrates context‐dependent temporal changes in hypnotic agents involved in suicide attempts, as identified from empty medication packages collected at presentation. Across the 5‐year study period (April 2020–March 2025; labeled as 2021–2025 by the ending year), involvement of orexin receptor antagonists and melatonin receptor agonists (OMs) increased significantly over time when analyses were conducted using either all suicide attempts or overdose‐related suicide attempts as denominators. In contrast, a significant decrease in benzodiazepine receptor agonist (BZ) involvement was detectable only when analyses were restricted to overdose‐related suicide attempts. Together, these findings suggest that non‐GABAergic hypnotics are increasingly represented among overdose‐related suicide attempt presentations, whereas reductions in BZ involvement are selectively concentrated in overdose‐related contexts.

The steady rise in OM involvement likely mirrors increasing availability of DORAs/MRAs in routine insomnia care and growing confidence in their benefit–risk profile, alongside guideline recommendations that emphasize safety in high‐risk populations [5, 6, 7, 8]. Notably, our supplementary breakdown suggests that the observed increase in OMs was largely attributable to DORAs rather than MRAs (ramelteon), which remained uncommon (Table S1; Figure S2). From a suicide prevention perspective, medication selection and access can be viewed as components of means safety: because self‐poisoning methods are partly determined by medications available at home, choosing hypnotics with lower overdose toxicity and lower abuse liability may reduce the medical severity of future episodes even when suicidal crises recur. This rationale is supported by evidence that benzodiazepines vary in relative toxicity and can be associated with severe outcomes in overdose [2, 3, 4], whereas non‐GABAergic hypnotics are generally considered less likely to cause profound respiratory depression when taken alone [5, 6, 7]. Cochran–Armitage tests supported this component pattern: DORA involvement increased significantly over time (*p* for trend < 0.01 in both denominator settings), whereas MRA involvement did not (*p* for trend > 0.3).

Why, then, was a temporal decline in BZ involvement apparent only in overdose‐related attempts? Overdose presentations are particularly sensitive to changes in medication access: the substances used in self‐poisoning often mirror what is available at home, and shifts in insomnia treatment (including increased use of DORAs/MRAs and broader efforts in clinical practice to limit benzodiazepine exposure) may alter the mix of hypnotics involved in overdoses over time. In contrast, when all suicide attempts are pooled, the denominator includes methods in which medication access is not central, diluting overdose‐specific changes. Our denominator‐specific approach underscores how analytic framing can mask or reveal clinically meaningful shifts, and it may be applicable to other pharmacoepidemiologic questions in suicide‐related care. Notably, our study ascertained medications involved from empty packages and did not directly measure prescribing decisions or dispensing quantities.

Clinically, these findings support a risk‐sensitive approach to hypnotic selection after suicide attempts. For patients with insomnia and elevated suicide risk—particularly those with recent or recurrent overdose—switching from benzodiazepines to DORAs/MRAs may be one practical strategy to mitigate overdose toxicity while maintaining symptom control [5, 6, 7, 8]. However, pharmacological harm reduction cannot replace comprehensive suicide prevention: limiting dispensed quantities, reviewing polypharmacy, addressing comorbid substance use, and providing evidence‐based treatments for underlying psychiatric disorders remain essential. Moreover, OMs are not risk‐free; excessive sedation, falls, and next‐day impairment may occur, especially in older adults or in the presence of drug interactions, underscoring the need for careful monitoring even when a drug is considered safer in overdose.

Several limitations warrant consideration. First, this retrospective study was conducted at three hospitals in Japan (Dokkyo Medical University Hospital, Jichi Medical University Hospital, and Hachinohe City Hospital), and patterns of medications involved in suicide attempts may reflect local practice, formulary availability, and temporal changes in referral pathways. Second, we did not have toxicology‐confirmed data on the specific agents ingested, co‐ingestants, or overdose severity; medication identification relied on empty packages and routine clinical documentation, which may be incomplete, and the ingested dose cannot be confirmed. Third, we focused on BZs and non‐GABAergic hypnotics (DORAs/MRAs) and did not evaluate other agents commonly involved in self‐poisoning (e.g., Z‐drugs analyzed separately from BZs in some settings, sedating antidepressants, antipsychotics, analgesics, or cardiovascular medications) that may also contribute to self‐poisoning. Fourth, the trend tests were unadjusted, and residual confounding by changes in case‐mix over time is possible. Larger multicenter studies linking exposure ascertainment to toxicology and clinical outcomes (e.g., need for airway support, ICU admission, length of stay, and repeated suicide attempts) are needed to clarify whether shifts from BZs toward DORAs translate into measurable reductions in overdose morbidity. Nevertheless, our findings provide real‐world evidence that the hypnotic profile of suicide attempt presentations is evolving in a nuanced, context‐dependent manner. Finally, although the component analyses suggested that the OM increase was driven primarily by DORAs and that MRA involvement did not change significantly over time, the small number of MRAs limits precision and warrants cautious interpretation.

## Author Contributions

N.Y.‐F. conceived and designed the study. T.S., H.I., S.S., and N.Y.‐F. collected the data. N.S., M.F., H.I., and S.S. supervised the project. N.Y.‐F. and N.S. performed the statistical analyses and drafted the manuscript. All authors critically reviewed the manuscript and approved the final version.

## Funding

The authors have nothing to report.

## Ethics Statement

This multicenter retrospective cohort study was centrally reviewed and approved by the Ethics Committee of Dokkyo Medical University. All participating institutions relied on this central approval. Given the retrospective nature of the study, the requirement for written informed consent was waived, and an opt‐out approach was used.

## Conflicts of Interest

The authors declare no conflicts of interest.

## Supporting information


**Figure S1:** Annual numbers of suicide attempt encounters (all methods) and overdose‐related suicide attempt encounters across the study period.

**Figure S2:** Annual counts of dual orexin receptor antagonist (DORA) and melatonin receptor agonist (MRA; ramelteon) agents identified from empty medication packages.

**Table S1:** Annual counts of dual orexin receptor antagonist (DORA) and melatonin receptor agonist (MRA; ramelteon) agents identified from empty medication packages. Counts are summarized at the agent level and may exceed the number of OM‐involved encounters.

## Data Availability

The data that support the findings of this study are not publicly available because they contain sensitive suicide‐related clinical information and, given the multicenter design and relatively small annual cell sizes, de‐identified records may still carry a risk of re‐identification. The dataset may be made available from the corresponding author upon reasonable request, subject to approval by the relevant ethics committees and the participating institutions, and the completion of a data‐sharing agreement where required.
