# Supplementary figures and images for: Context‐Dependent Temporal Changes in Hypnotics Involved in Suicide Attempts
Source: Neuropsychopharmacol Rep. 2026 Apr 30;46(2):e70126. doi: 10.1002/npr2.70126 (PMC13129489; doi:10.1002/npr2.70126)

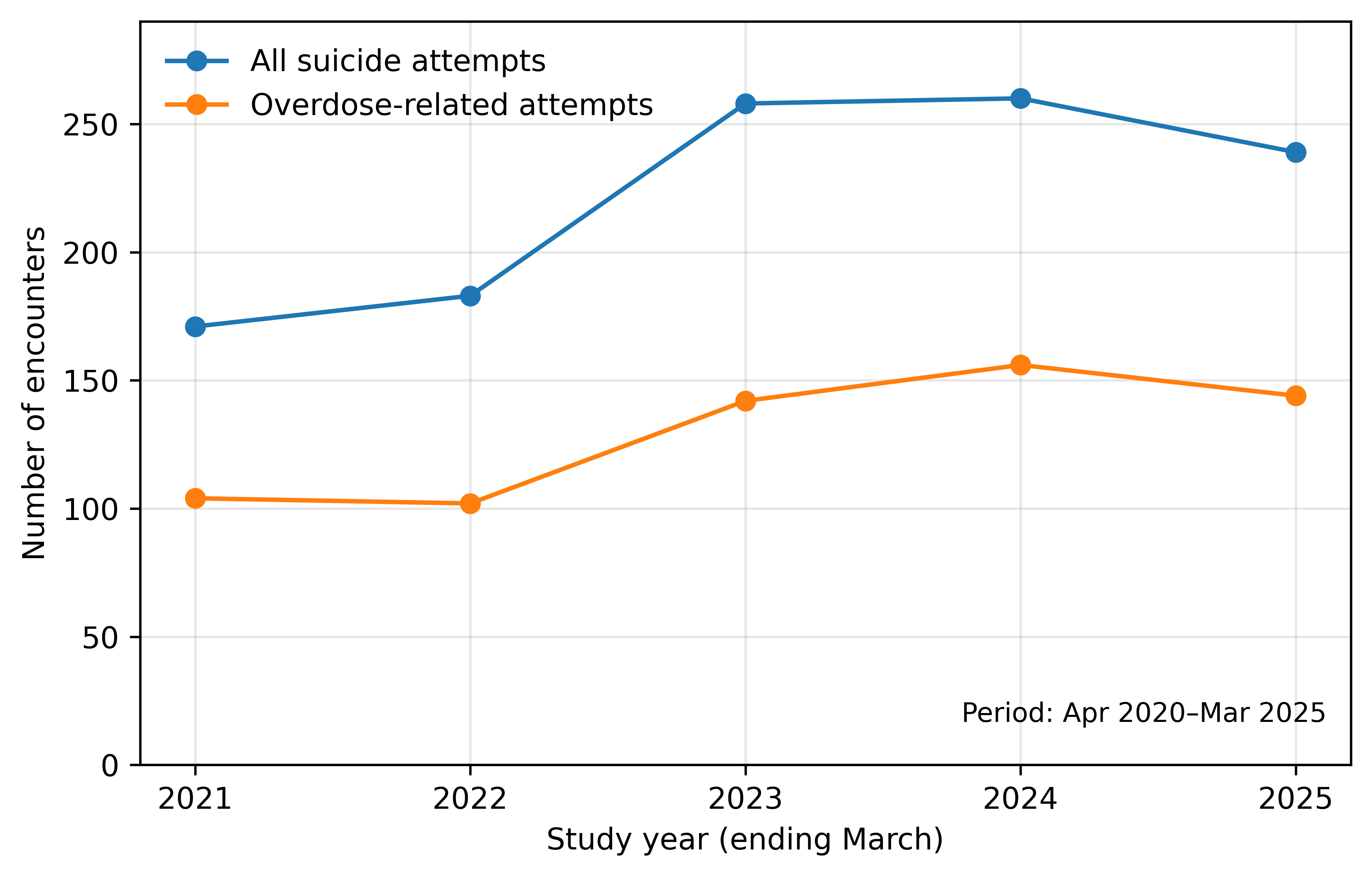

Supplement: Supplementary file 1 — Figure S1: Annual numbers of suicide attempt encounters (all methods) and overdose‐related suicide attempt encounters across the study period. [file NPR2-46-e70126-s002.tiff]

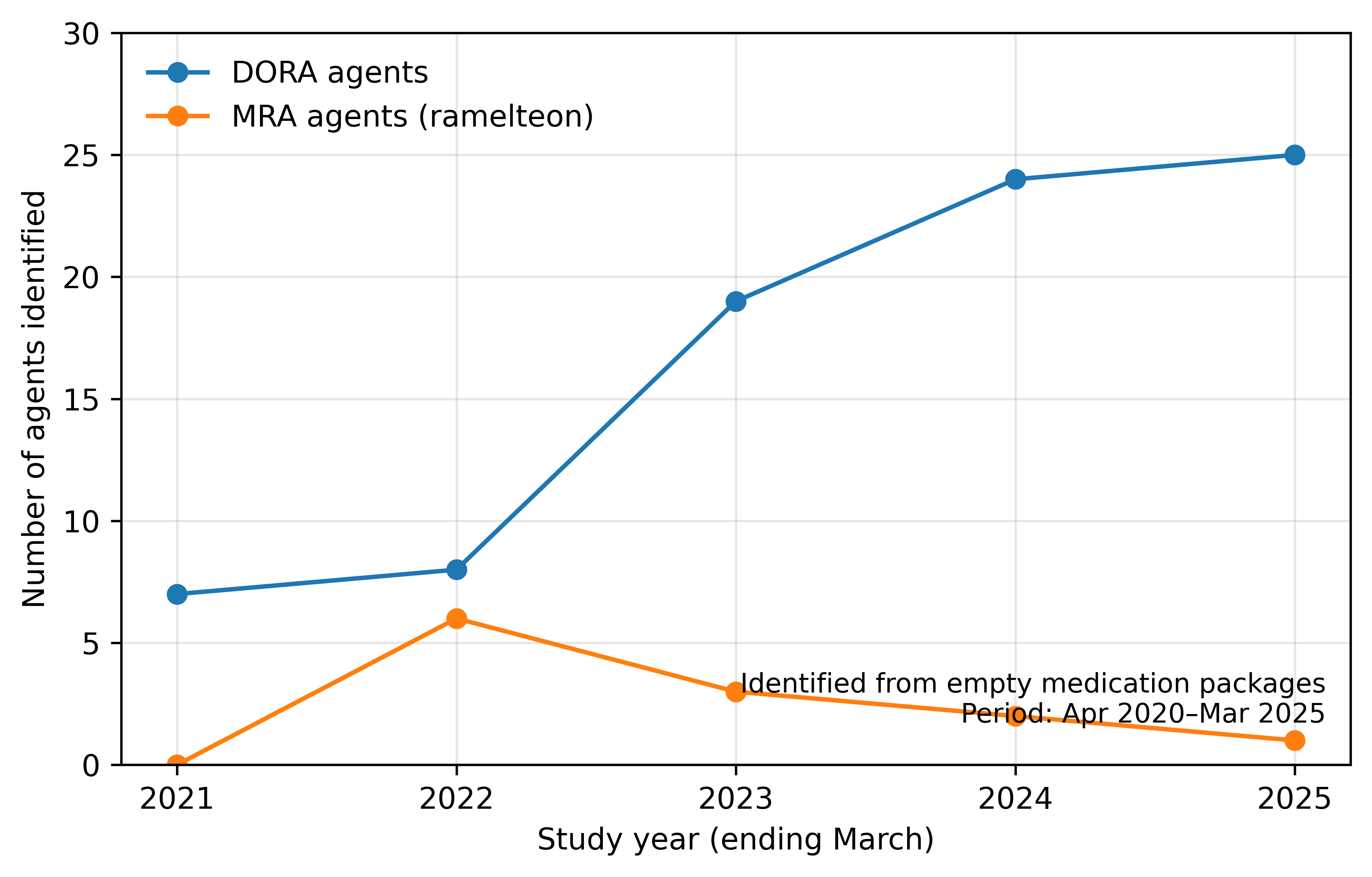

Supplement: Supplementary file 2 — Figure S2: Annual counts of dual orexin receptor antagonist (DORA) and melatonin receptor agonist (MRA; ramelteon) agents identified from empty medication packages. [file NPR2-46-e70126-s003.tiff]
